# Evaluating the Influence of Spray and Immersion Disinfection on the Dimensional Accuracy of Impression Materials: A Comprehensive Review

**DOI:** 10.7759/cureus.88921

**Published:** 2025-07-28

**Authors:** Abhilasha Masih, Ranjeet R Gandagule, Ankita Tiwari, Elampavai Elangovan, Elanangai E, Vivek Choukse, Nikita Saini

**Affiliations:** 1 Department of Prosthodontics and Crown and Bridge, Dr. Hedgewar Smruti Rugna Seva Mandal's Dental College and Hospital, Hingoli, IND; 2 Department of Prosthodontics and Crown and Bridge, Shri Balaji Institute of Dental Sciences, Raipur, IND; 3 Department of Oral Pathology and Microbiology, Sri Venkateshwara Dental College and Hospital, Bengaluru, IND; 4 Department of Prosthodontics and Implantology, Sri Sai Lakshmi Dental Clinics, Bangalore, IND; 5 Department of Pedodontics and Preventive Dentistry, Hitkarini Dental College and Hospital, Jabalpur, IND

**Keywords:** dental infection control, dimensional stability, immersion disinfection, impression materials, prosthodontics, spray disinfection

## Abstract

Effective infection control in dental practice is essential, particularly during impression-making procedures that risk transmitting pathogens such as HIV and hepatitis B. This review evaluates how spray and immersion disinfection methods affect the dimensional accuracy of impression materials. A comprehensive search of electronic databases identified studies assessing the impact of these disinfection techniques on various materials. The findings indicate that elastomeric materials such as addition and condensation silicones and polysulfides, maintain dimensional stability with both methods. In contrast, hydrocolloids such as alginate and polyether are more prone to distortion, especially with prolonged immersion in sodium hypochlorite or glutaraldehyde. Spray disinfection, when applied for short durations, minimizes dimensional changes and is preferred for hydrocolloids. Glutaraldehyde, particularly in spray form, proved more compatible than sodium hypochlorite. The review emphasizes that disinfection protocols should be material-specific, balancing microbial control with preservation of impression accuracy. Standardized guidelines are needed to ensure optimal clinical outcomes.

## Introduction and background

Ensuring infection control in dental practice is paramount, as the oral cavity harbors a diverse range of microorganisms, some of which can pose serious health risks. One often overlooked source of cross-contamination is impression-making, a fundamental procedure in dentistry [1]. These materials come into direct contact with saliva and blood, enabling the transmission of pathogens if not disinfected appropriately [1,2]. Notably, they may serve as vectors for infections such as hepatitis B, hepatitis C, HIV, and tuberculosis [1]. Microorganisms can persist on impression surfaces and subsequently transfer to gypsum casts, thereby increasing the potential for indirect disease transmission [2]. Simply rinsing impressions under running water is insufficient, as many viruses and bacteria adhere tightly to the material surface. This washing step typically removes only 40% to 90% of microbial contaminants, making it a preparatory measure rather than a definitive decontamination step [3].

To mitigate this risk, the American Dental Association (ADA) recommends disinfecting impressions immediately after their removal from the oral cavity, a step that helps prevent cross-contamination and protects not only patients but also dental healthcare providers, including assistants and laboratory personnel [4]. The physical and chemical properties of impression materials, such as hydrophilicity (ability to interact with water), surfactant incorporation (agents that improve wettability), and resistance to fluid exposure, significantly influence their clinical performance and interaction with disinfectants [5]. Importantly, these properties also affect dimensional stability, which is critical to the accuracy of dental restorations. Materials such as alginate (a hydrocolloid) and elastomers (e.g., addition silicones, condensation silicones, and polyethers) respond differently to disinfectant exposure [6].

While infection control is non-negotiable, improper disinfection may compromise the dimensional integrity of impressions. Among various methods, spray and immersion disinfection are most commonly employed due to their simplicity and effectiveness. However, their interaction with different impression materials can lead to inconsistencies in accuracy and surface detail reproduction. Current protocols often lack material-specific guidance, raising concerns about either insufficient sterilization or distortion of results. This review critically examines the influence of spray and immersion disinfection methods on the dimensional accuracy of commonly used dental impression materials. By evaluating this trade-off, it aims to support a more evidence-based approach to achieving both infection control and clinical precision.

Materials and methods

A structured literature search was conducted to identify studies evaluating the effect of spray and immersion disinfection methods on the dimensional stability of dental impression materials. The search spanned publications from September 1986 to January 2024 and included databases such as PubMed/Medical Literature Analysis and Retrieval System Online (MEDLINE), Scopus, Cochrane Library, EBSCO, and Google Scholar. The search terms used were “impression materials” AND (“spray disinfection” OR “immersion disinfection”) AND (“dimensional stability” OR “dimensional accuracy”). No filters were applied regarding study type, and both in vitro and clinical studies were considered. Additional relevant publications were located through a manual screening of the reference lists from the initially selected articles.

Inclusion and Exclusion Criteria

The inclusion criteria for this review encompassed studies published in English that investigated the dimensional stability of widely used impression materials such as alginate, polyether, polysulfide, condensation silicone, addition silicone, zinc oxide eugenol, agar, or polyvinyl ether siloxane (PVES) following disinfection. The studies had to use spray and/or immersion techniques with commonly used chemical agents such as sodium hypochlorite, glutaraldehyde, iodophor, phenolics, or chlorhexidine. The focus was specifically on those that examined the impact of exposure durations and concentrations on the physical or dimensional integrity of the impression material. Articles were excluded if they did not report dimensional changes, involved only alternative disinfection modalities (e.g., microwave or ultraviolet methods), or lacked sufficient methodological detail. Non-English publications and duplicates were also excluded.

**Figure 1:**
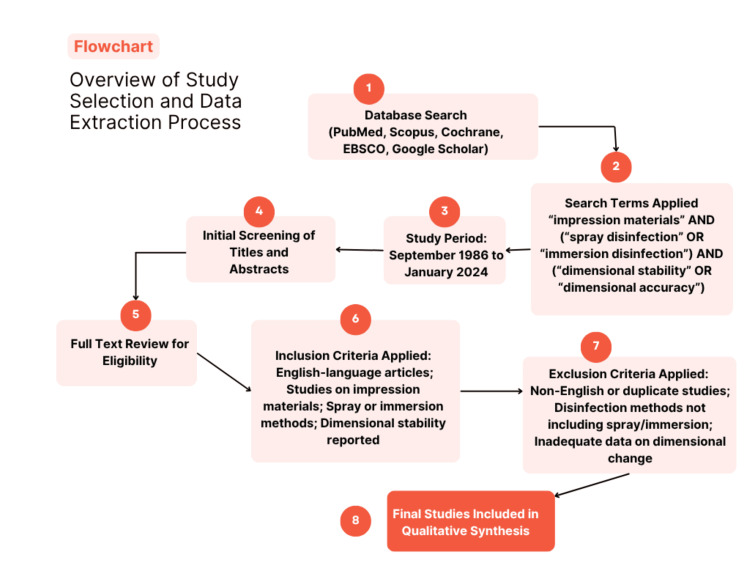
Overview of study selection and data extraction process This figure has been created by the author

Two reviewers independently screened and selected the studies, extracting relevant information, including authorship, year of publication, type of impression material evaluated, disinfection method used, and the observed dimensional changes, which collectively contributed to drawing a well-supported conclusion. Inconsistencies were resolved through discussion. Due to methodological heterogeneity among studies, particularly in terms of disinfectant types, concentrations, exposure times, and evaluation techniques, a quantitative meta-analysis was not performed. Instead, findings were synthesized descriptively, focusing on patterns and consistencies across the included literature. An overview of the study selection and data extraction process is displayed in Figure 1.

## Review

Overview of impression materials in prosthodontics

**Figure 2:**
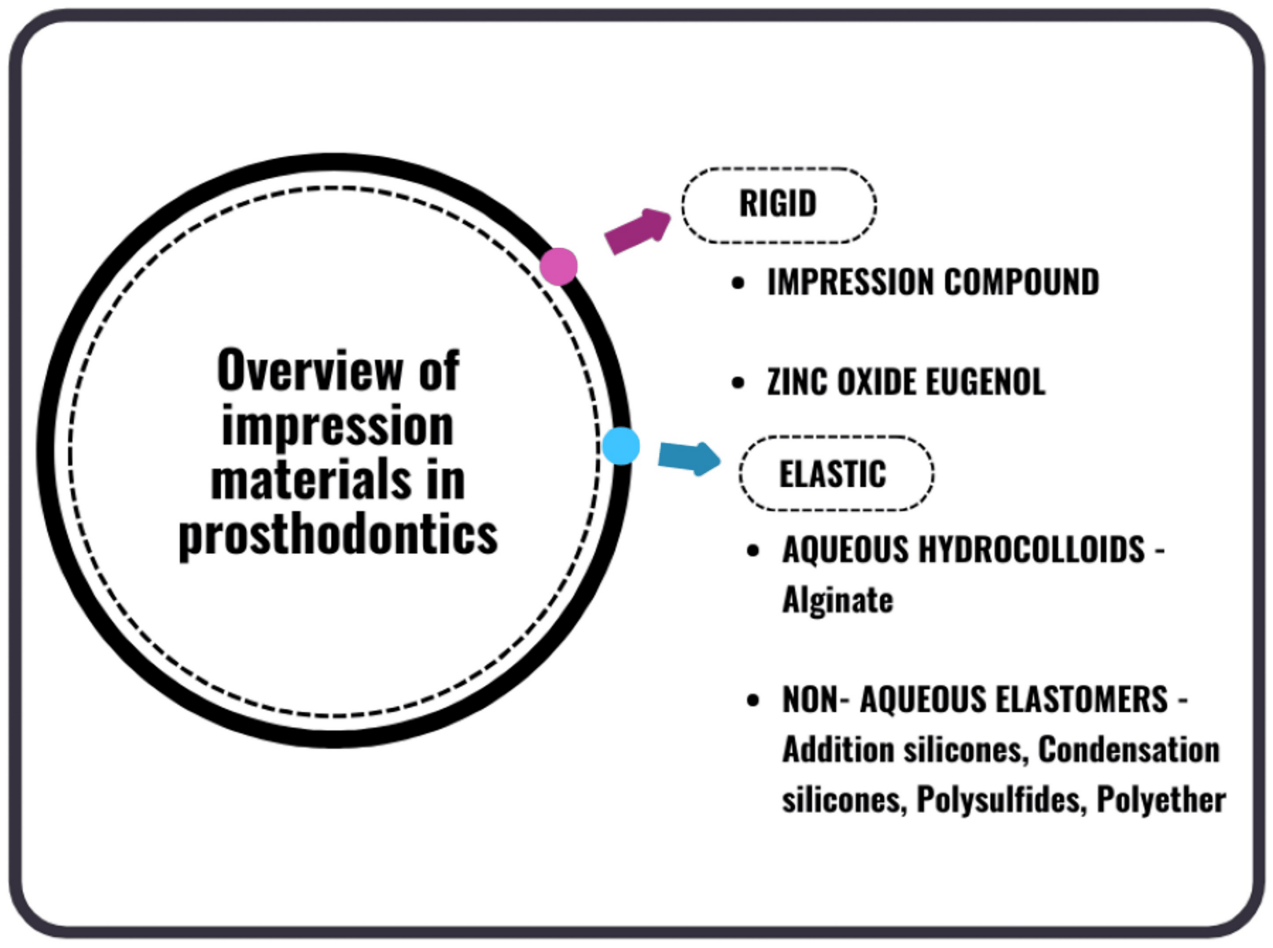
Key impression materials in dental practice This figure has been created by the author

Impression materials are used to capture the morphology of teeth and surrounding oral structures, producing a dental impression that is subsequently poured with dental plaster to create a cast. This process generates a precise three-dimensional replica of the oral cavity, enabling dental procedures to be performed accurately, even in the patient's absence [7]. Dental impression materials are broadly categorized into rigid (nonelastic) and elastic materials. In prosthodontics, elastic impression materials are predominantly used due to their ability to accurately capture fine details and adapt to undercuts [4]. The key impression materials commonly used are depicted in Figure 2.

Disinfection of impression materials

The ADA Council (1996) and the Fédération Dentaire Internationale (FDI) (1998) recommend disinfecting impression materials using immersion or spray methods for two to three minutes. Similarly, the Centers for Disease Control and Prevention (CDC) supports these disinfection techniques, emphasizing the use of appropriate disinfectant solutions to prevent cross-contamination in dental practice [8,9]. A critical factor in impression disinfection is dimensional stability, which refers to a material's ability to retain its original size and shape. This property is essential for ensuring the accurate replication of dies and the fabrication of precise prostheses [10]. However, disinfectants can induce chemical or physical reactions with impression materials, potentially compromising their integrity. An ideal disinfectant and the process used must effectively eliminate microbes while preserving the dimensional accuracy and surface details of both the impression and the resulting model. Striking this balance is crucial to maintaining infection control without compromising clinical precision in prosthodontic procedures [9].

Immersion and spraying method for disinfecting impression materials

At present, no universally established protocol exists for disinfecting dental impressions. However, immersion disinfection is widely acknowledged for its effectiveness in minimizing cross-contamination risks [11]. Since chemical disinfectants primarily act on the surface, pre-cleaning with brushing and rinsing is advisable, as rinsing alone may not sufficiently eliminate contaminants like blood and saliva. Among the available disinfection methods, immersion is often favored, as it provides complete coverage of both the impression material and tray, ensuring a more thorough and dependable disinfection process [12]. 

A highly effective method for disinfecting impressions is to immerse them in a disinfectant solution for 30 minutes. This ensures that most hydrophobic impression materials maintain their original shape with negligible distortion throughout the process [13]. Compared to spraying, this technique reduces the risk of incomplete coverage and minimizes disinfectant inhalation hazards for the user. However, spraying is also regarded as an effective disinfection method, as it helps prevent impression distortion, which can occur with prolonged immersion [12].

The effects of disinfection through spraying or immersion on the dimensional stability of various impression materials have been studied in this review. A summary of these findings is provided in Table 1.

**Table 1:** Summary of studies included in this qualitative synthesis

Serial Number	Author and year of publication	Relevant conclusion
1	Hemalatha R et al. (2016) [6]	Immersion disinfection ensures better surface coverage and prolonged exposure, enhancing microbial reduction. However, hydrophilic materials such as alginate and polyether show dimensional distortion due to water absorption when immersed for extended durations. Spray disinfection, though less thorough, preserves dimensional accuracy and is preferred for these materials. Agar and zinc oxide eugenol (ZOE) show acceptable immersion stability within the recommended times. Elastomeric materials like addition silicone and polysulfide can withstand immersion, but prolonged exposure, even in glutaraldehyde, may affect polyether.
2	Pal PK et al. (2014) [8]	Immersion disinfection of elastomeric impressions in 2% glutaraldehyde or sodium hypochlorite (1% and 4%) effectively eliminates microbes without compromising surface detail. Among these, 1% sodium hypochlorite provided superior surface detail reproduction.
3	Wezgowiec J et al. (2022) [11]	Disinfection significantly reduced the hardness of light-bodied C-silicone (Oranwash L), while other properties remained largely unaffected. Spray disinfection is recommended for Oranwash L. For other silicones, all disinfection methods preserved dimensional stability and material properties
4	Herrera SP et al. (1986) [14]	Short-term immersion in 0.5% or 1% sodium hypochlorite, glutaraldehydes, povidone-iodine, or halogenated phenol does not significantly affect the dimensional accuracy of rubber impression materials. Immersion disinfection is suitable for rubber base and vinyl polysiloxane impressions used in study models and removable prostheses.
5	Ivanis T et al. (2000) [15]	Immersion of polyether in chlorhexidine was found inappropriate due to adverse effects on material properties.
6	Silva SM et al. (2004) [16]	Immersion of condensation silicone in 1% sodium hypochlorite and 2% glutaraldehyde for 10 or 20 minutes did not cause dimensional changes; 24-hour immersion of silicone in chlorhexidine was also acceptable.
7	Walker MP et al. (2007) [17]	Vinyl polysiloxane and polyether showed dimensional changes when immersed in 0.5% sodium hypochlorite or phenol for 10 minutes or one hour, but these changes were within American Dental Association (ADA) limits. Longer exposure caused surface deterioration in polyether.
8	Kotsiomiti E et al. (2008) [18]	Immersion is more effective than spray for hydrocolloids, but must be time-limited to prevent distortion. Polyethers can be safely disinfected by spray, though modern types also tolerate immersion. Hydrophilic silicones may absorb disinfectants during prolonged immersion, risking dimensional changes. Hydrophobic elastomers remain stable even with extended immersion.
9	Melilli D et al. (2008) [19]	Immersion disinfection of polyether (PE) and addition silicone (polyvinyl siloxane (PVS)) in Sterigum Powder (SP) and MD520 resulted in dimensional changes ≤0.5%, which are not clinically significant. Although statistically significant differences were observed across time intervals, especially with SP, the changes remained within ADA-acceptable limits, indicating that immersion in these disinfectants is safe for both materials.
10	Amin WM et al. (2009) [20]	A 10-minute immersion in 0.5% sodium hypochlorite was recommended for disinfection of alginate, addition silicone, condensation silicone, and ZOE impression materials.
11	Bustos J et al. (2010) [21]	Five-minute immersion of alginate and silicone impression materials in 0.5% sodium hypochlorite or 2% glutaraldehyde was effective with minimal impact on dimensional stability.
12	Saber FS et al. (2010) [22]	Spray atomization disinfection of condensation silicone impressions (Spidex® and Rapid®) with 5.25% sodium hypochlorite and 10% iodophor caused significant dimensional changes. However, these changes were not substantial enough to critically affect the accuracy of fixed partial denture restorations.
13	Rad FH et al. (2010) [23]	Spray disinfection is the most commonly used method for hydrophilic impression materials such as hydrocolloids (e.g., alginate), while immersion is not advised due to dimensional changes.
14	Carvalhal CI et al. (2011) [24]	Immersion of addition silicone, polyether, and polysulfide in 2% glutaraldehyde for 5 or 10 minutes caused no dimensional changes. However, immersion for 20 minutes resulted in distortion, highlighting the influence of immersion time on dimensional stability.
15	Muzaffar D et al. (2012) [25]	Continuous shrinkage was observed in alginate materials (Blueprint Cremix and Hydrogum) during immersion in distilled water and disinfectants (Perform ID and sodium hypochlorite). Dimensional changes increased with time and were influenced by material thickness, with notable distortion seen after 10 minutes, especially in thinner samples.
16	Shetty S et al. (2013) [26]	A 10-minute immersion time is recommended for the disinfection of various impression materials to balance efficacy and dimensional stability.
17	Guler U et al. (2013) [27]	A 30-minute immersion was recommended for disinfecting polyether impression material.
18	Ahila SC et al. (2014) [28]	Condensation and addition silicone impression materials showed insignificant dimensional changes after 10-minute spray or immersion disinfection.
19	Duseja S et al. (2014) [29]	Addition silicone remained dimensionally stable after 10 minutes to 1 hour of immersion in different disinfectants, but polyether showed significant dimensional changes even at 10 minutes.
20	Nassar U et al. (2015) [30]	Immersion in 2.5% glutaraldehyde maintained dimensional stability of both addition and condensation silicones within ADA limits, with addition silicone showing superior stability.
21	Demajo JK et al. (2016) [31]	Spray disinfection with glutaraldehyde effectively eliminates microbes from addition silicone and alginate impressions without affecting dimensional stability.
22	Ismail HA et al. (2017) [32]	ZOE impressions maintained dimensional stability after immersion in 1% sodium hypochlorite and 2% glutaraldehyde for up to 60 minutes. For alginate, immersion should be limited to 10 minutes to avoid dimensional distortion.
23	Babiker GH et al. (2018) [33]	Alginate impressions showed insignificant dimensional changes when sprayed with 1% and 5.25% sodium hypochlorite, but significant distortion occurred with immersion.
24	Alzain S (2020) [34]	Spray disinfection of polyether and vinyl polysiloxane with 0.5% glutaraldehyde for 10 minutes improved their wettability, as glutaraldehyde acted as a surface-reducing agent, enhancing the wetting potential of the impression materials.
25	Hassan SAB et al. (2023) [35]	Polyvinyl ether siloxane (PVES) impressions showed no significant dimensional changes after 10-minute immersion in disinfectants. However, sodium hypochlorite caused clinically significant changes (p = 0.049), while 2%–2.5% glutaraldehyde had no adverse effect on dimensional stability.
26	Chidambaram RS et al. (2024) [36]	Although the ADA recommends immersion disinfection for alginate impressions, studies have shown significant dimensional changes with immersion. Therefore, spray disinfection is preferred to preserve the dimensional stability of irreversible hydrocolloid materials.
27	Fransiska A et al. (2024) [37]	Spraying alginate impressions with 5.25% sodium hypochlorite caused dimensional changes that increased with longer disinfection times. The least distortion occurred at 5 minutes, and the most at 15 minutes, indicating a time-dependent impact on dimensional stability.

Study evaluation

The studies analyzed in the table provide a comprehensive evaluation of the effects of immersion and spray disinfection on the dimensional stability of various impression materials.

Dental impressions, being potential carriers of pathogens, require effective disinfection protocols. Studies have shown that impressions rinsed with water alone still harbored microbial growth in about 77% of cases. Therefore, current disinfection strategies include either spray disinfection followed by sealing in a bag or immersion disinfection. While the spray technique demonstrates similar antimicrobial effectiveness to immersion, it is generally considered safer for dimensional accuracy due to minimal liquid absorption. Immersion, in contrast, offers thorough coverage and prolonged contact time, making it more efficient microbiologically, though it poses a risk of dimensional instability, particularly in hydrophilic materials such as alginate and polyether [38]. This concern was clearly supported by Hemalatha R et al. (2016), who reported that although immersion offered greater microbial reduction, hydrophilic materials like alginate and polyether exhibited distortion when immersed for longer durations. Spray disinfection, while less penetrative, better preserved dimensional accuracy in such materials [6]. A similar conclusion was drawn by Rad FH et al. (2010), who favored spray disinfection for hydrocolloids, noting that immersion often resulted in dimensional instability [23]. However, immersion disinfection remains the preferred method for elastomeric materials like addition silicones and polysulfides, which are hydrophobic and less prone to water uptake. Pal PK et al. (2014) demonstrated that immersion in 2% glutaraldehyde or sodium hypochlorite (1% and 4%) effectively disinfected elastomeric impressions without compromising surface detail, with 1% sodium hypochlorite showing superior reproduction fidelity [8]. Similarly, Carvalhal CI et al. (2011) noted that immersion of additional silicone, polyether, and polysulfide in 2% glutaraldehyde for up to 10 minutes did not induce dimensional distortion, though immersion beyond 20 minutes did [24].

Muzaffar D et al. (2012) reinforced the role of immersion time in influencing distortion. They observed that alginate materials showed progressive shrinkage with increasing immersion time, and thinner specimens were more affected, suggesting that material thickness and exposure duration are critical variables [25]. On the other hand, Melilli D et al. (2008) tested polyether and addition silicone in Sterigum powder and MD520, and although statistically significant differences were seen at various time points, all changes remained within clinically acceptable limits (≤0.5%) [19]. For specific materials, Walker MP et al. (2007) reported that polyether and vinyl polysiloxane showed dimensional changes on immersion in sodium hypochlorite and phenol for both 10 minutes and 1 hour. Although these changes were within ADA thresholds, prolonged exposure led to deterioration, particularly in polyether [17]. In contrast, Silva SM et al. (2004) found that immersion of condensation silicones in sodium hypochlorite and glutaraldehyde for 10-20 minutes caused no significant changes, and even a 24-hour immersion in chlorhexidine was deemed safe [16].

Spray disinfection remains particularly relevant in alginate and hydrocolloid impressions. Babiker GH et al. (2018) demonstrated that while immersion caused significant dimensional changes, spray disinfection using 1% and 5.25% sodium hypochlorite maintained dimensional integrity [33]. Fransiska A et al. (2024) elaborated further, showing that distortion increased with longer spray times, recommending five minutes as optimal to limit deformation [37]. Saber FS et al. (2010) evaluated spray disinfection on condensation silicones and found that while statistically significant dimensional changes occurred, these did not compromise the clinical accuracy of fixed prosthesis fabrication [22]. Ahila SC et al. (2014) similarly noted that both spray and immersion caused insignificant changes in addition and condensation silicones over 10 minutes of exposure [28]. Some studies have explored the surface and mechanical effects of disinfection methods. Wezgowiec J et al. (2022) found that while spray and immersion preserved dimensional stability in various silicones, light-bodied C-silicone (Oranwash L) showed reduced hardness post disinfection, leading to a preference for spray in that material [11]. Alzain S (2020) found that spray disinfection with glutaraldehyde enhanced the wettability of polyether and vinyl polysiloxane, a favorable trait for impression performance [34].

When comparing disinfectant agents, Nassar U et al. (2015) confirmed that glutaraldehyde at 2.5% better preserved the dimensional stability of addition and condensation silicones compared to other agents [30]. Hassan SAB et al. (2023) also found that sodium hypochlorite caused clinically significant changes in PVES, while glutaraldehyde had no such adverse effects [35]. Despite the ADA recommending immersion for alginates, practical findings support the superiority of spray disinfection for preserving dimensional accuracy. Chidambaram RS et al. (2024) emphasized this point, citing significant distortion from immersion in alginate and advocating for spray as the preferred technique [36]. The rationale behind these differences can be attributed to the nature of the materials. For instance, the pooling effect in spray disinfection creates localized action, while immersion allows disinfectant to penetrate uniformly. However, this can be detrimental in hydrophilic materials, as internal penetration has been noted in alginate up to 3 mm and blood infiltration to about 0.7-2 mm from the surface [38]. This highlights the critical balance between achieving effective disinfection and maintaining dimensional fidelity, which must be guided by material properties, type of disinfectant, and duration of exposure.

Comparative features of spray and immersion disinfection of impression materials, based on this literature review, are summarized in Table 2.

**Table 2:** Comparative overview: spray vs. immersion disinfection

Feature	Spray disinfection	Immersion disinfection
Surface coverage	Provides localized surface disinfection; risk of incomplete coverage due to pooling and uneven distribution, especially in undercut or internal areas [6,23,38].	Offers comprehensive surface disinfection due to complete submersion and prolonged contact time, ensuring uniform disinfectant exposure [6,8].
Disinfection efficacy	Effective for routine microbial control but may miss internal contamination or deep penetration in materials like alginate [23,38].	Considered more reliable due to extended contact; shown to be more thorough in microbial elimination [8,14,38].
Dimensional stability	Superior in hydrophilic materials like alginate and polyether; minimal distortion when used appropriately [6,23,36].	Risk of dimensional distortion, especially in hydrocolloids and polyether, due to water absorption during immersion [6,18,25,36].
Material compatibility	Recommended for alginate, polyether, and hydrocolloids where dimensional stability is a priority [6,33,36].	Best suited for elastomeric materials like addition silicone, condensation silicone, and polysulfide, which can tolerate immersion [8,16,28].
Time sensitivity	Less time-sensitive; minor changes observed even at 10–15 minutes [28,37].	Highly time-dependent; distortion increases with longer immersion, especially beyond 10–20 minutes [15,24,25].
Surface detail preservation	Preserves surface detail in hydrophilic and light-bodied silicones (e.g., Oranwash L) [11,31].	Generally maintains surface detail in elastomeric materials unless immersion exceeds safe limits [8,14,30].
Limitations	Pooling leads to localized action; less effective against internal or embedded pathogens [23,38].	Potential material swelling, leaching, or distortion with prolonged exposure or incompatible disinfectants (e.g., chlorhexidine with polyether) [15,17,25].
Clinical recommendation	Preferred for chairside use and for materials sensitive to water uptake; safer for maintaining impression accuracy [6,33,36].	Recommended when comprehensive disinfection is essential and the material tolerates immersion without dimensional changes [14,22].

Based on the literature used in this study, the material-wise effect of spray and immersion disinfection on dimensional stability has been displayed in Table 3.

**Table 3:** Material-wise effect of spray and immersion disinfection on dimensional stability

Material Type	Disinfection method	Effect on dimensional stability
Alginate, polyether, and elastomers	Spray versus immersion	Spray preferred for hydrophilic materials due to less distortion; immersion was suitable for elastomers [6].
Elastomers	Immersion	No compromise in dimensional stability; 1% sodium hypochlorite gave the best results [8].
Light-bodied C-silicone	Spray versus immersion	Spray recommended for Oranwash L; immersion reduced hardness [11].
Rubber impression materials	Immersion	No significant effect on dimensional accuracy [14].
Polyether	Immersion (chlorhexidine)	Caused adverse effects; immersion was not suitable [15].
Condensation silicone	Immersion	No dimensional changes at 10–20 minutes; safe for short-term immersion [16].
Vinyl polysiloxane and polyether	Immersion	Dimensional changes occurred but remained within the American Dental Association (ADA) limits [17].
Hydrocolloids, polyether, and silicones	Spray versus immersion	Immersion was more effective but caused distortion in hydrophilic materials; elastomers remained stable [18].
Polyether and polyvinyl siloxane (PVS)	Immersion	Dimensional changes ≤0.5%; clinically insignificant [19].
Alginate and silicone	Immersion	Minimal impact on dimensional stability with five-minute immersion [21].
Condensation silicone (Spidex®, Rapid®)	Spray	Caused dimensional changes, but not clinically significant [22].
Alginate	Spray	Preferred over immersion to avoid dimensional distortion [23].
Addition silicone, polyether, and polysulfide	Immersion	No changes at five to 10 minutes; distortion noted after 20 minutes [24].
Alginate	Immersion	Shrinkage increased with time; more distortion in thinner samples [25].
Condensation and addition silicones	Spray and immersion	Both methods caused insignificant changes [28].
Addition silicone and polyether	Immersion	Silicone stable; polyether showed distortion even after 10 min [29].
Addition and condensation silicones	Immersion	Stable within ADA limits; addition silicone was superior [30].
Zinc oxide eugenol (ZOE) and alginate	Immersion	ZOE stable; alginate should be limited to 10 minutes to avoid distortion [32].
Alginate	Spray versus immersion	Spray caused no significant change; immersion led to distortion [33].
Polyether and vinyl polysiloxane	Spray	Improved wettability; dimensional stability unaffected [34].
Polyvinyl ether siloxane (PVES)	Immersion	No change with glutaraldehyde; sodium hypochlorite caused slight clinical distortion [35].
Alginate	Spray versus immersion	Spray preferred due to significant changes seen with immersion [36].
Alginate	Spray	Time-dependent distortion noted; 5 min least, 15 min most distortion [37].

Clinical recommendations

Spray disinfection is preferable for hydrophilic materials such as alginate and polyether, as it minimizes dimensional distortion while maintaining acceptable antimicrobial efficacy [6,23,36]. A disinfection time of five to 10 minutes using 1% to 5.25% sodium hypochlorite spray is advised to limit dimensional changes [33,37]. Immersion disinfection is best suited for elastomeric materials like addition and condensation silicones, which maintain dimensional integrity when immersed in agents such as 2% glutaraldehyde or 0.5% sodium hypochlorite for short durations [8,16,24]. Selection of the disinfection method should therefore be tailored to the specific material, balancing infection control and accuracy.

Limitations of the study

While this review provides a comprehensive synthesis of available literature, variations across studies in disinfectant concentrations, exposure times, and brands of impression materials may influence result comparability. Additionally, only English-language studies were included, which may have limited the scope of evidence. Despite these constraints, the review offers valuable insights relevant to clinical decision-making and highlights consistent material-specific disinfection outcomes across multiple studies.

## Conclusions

The review highlights that both spray and immersion disinfection methods are viable, but their appropriateness depends on the chemical nature and hydrophilicity of the impression material. Spray disinfection, while less thorough in microbial elimination, is beneficial for preserving the dimensional accuracy of sensitive materials like alginate and polyether. Immersion, although potentially distorting for hydrophilic materials, remains the preferred method for elastomers due to better disinfection efficacy. A balanced, material-specific approach is essential for selecting an optimal disinfection protocol that ensures both infection control and clinical precision in dental prosthetic workflows.
